# Cardiac Proteomics

**DOI:** 10.1155/2014/903538

**Published:** 2014-06-15

**Authors:** Jatin G. Burniston, Anthony O. Gramolini, R. John Solaro

**Affiliations:** ^1^Muscle Physiology and Proteomics Laboratory, Research Institute for Sport and Exercise Sciences, Liverpool John Moores University Byrom Street, Liverpool, L3 3AF, UK; ^2^Department of Physiology, University of Toronto, Toronto, ON, Canada M5G 1L6; ^3^Department of Physiology and Biophysics, College of Medicine, University of Illinois at Chicago, Chicago, IL 60612-7342, USA

Approximately one-third of all deaths are attributed to cardiovascular diseases, and ischaemic heart disease is the primary cause of death globally. Hence there continues to be a pressing need for advances in preventative, diagnostic, and interventional strategies for cardiovascular disease. The multifactorial nature of cardiovascular disease, which encompasses both genetic and environmental factors, presents a major challenge to research in these areas. Furthermore, personalised strategies, which are anticipated to hold the key to reducing cardiovascular mortality rates, require yet more sophisticated biomarkers capable of accurately predicting disease risk, informing clinical decision-making, or monitoring therapeutic responsiveness.

Omic approaches, including genomics, transcriptomics, proteomics, and metabolomics, exploit genome information and use high-throughput analysis techniques. Because the data are comprehensive and the measurements are performed in parallel (i.e., reducing interassay variability), it lends itself to interrogation by bioinformatic tools that provide objective “unbiased”/“unsupervised” assessment of pertinent features, functional clusters, interactions, and regulatory networks. The marriage between comprehensive data collection and computational analysis is proven as powerful and pragmatic means of discovering new information and candidate biomarkers, which might not otherwise have been investigated. This editorial aims to orientate the reader with regard to where proteomic studies sit amongst the other levels of investigation (i.e., genomics, transcriptomics, etc.) and draw attention to the particular strengths and challenges involved in proteomic work.

The heritability of cardiovascular disease is estimated to be approximately 40%, but large multinational efforts to link disease risk to specific genetic variations have not yet identified high-penetrance genes with large effects. Indeed, the top 34 candidate genes explain less than 15% of the variance in heritable disease risk, and each gene has small (<5%) individual effects (reviewed in [1]). This emphasises the complex polygenetic nature of cardiovascular disease and also highlights the prominent influence that environmental factors have in determining cardiovascular risk. Because the genome is essentially static, genomic studies are not best placed to capture interactions with the environment. On the other hand, the transcriptome is cell-specific and can be influenced by epigenetic modifications and environmental inputs. Moreover, the technology for monitoring gene expression is particularly advanced and high-throughput arrays can be performed on a genome-wide scale, which provides an unsurpassed breadth of information.

Notwithstanding the strengths of transcriptome tools, gene expression (i.e., mRNA abundance) cannot be assumed to be associated with similar changes in the abundance or activity of their products (i.e., proteins). In part, this is because the net abundance of a protein is the result of the balance between its rates of synthesis and degradation and cannot be predicted solely from its mRNA transcript. In addition, post-transcriptional processing, microRNA interference and ribosomal capacity each affect the translation from mRNA to protein. Most important of all the activity of proteins, their cellular localisation and interaction with other proteins are regulated by a multitude of posttranslational modifications, including phosphorylation, acetylation, and glycosylation, which may combine to create numerous different “protein species” or “proteoforms” from each gene. Because of this, the number of proteins far exceeds the number of expressed genes and there is a wealth of information that can only be obtained by studying the proteome.

The protein complement of the heart directly underpins its functional properties, including electrophysiology, contractility, susceptibility to disease, and response to pathological insults. Moreover, the proteome is the interface between the environment and the genome. Signals from the environment are transduced through the proteome and may affect transcription factors, ribosomal capacity, or the activity of degradative processes to bring about changes (in the proteome) that alter the functional properties of the cell. The intimate link between cardiac physiology and the protein complement of the heart makes the proteome a highly fertile ground for discovering biomarkers to predict disease susceptibility, assist diagnoses, or help to monitor responses to therapeutic interventions. Indeed, the great strength of established prediction tools such as Framingham Risk Score lies in their ability to capture the interaction between an individual's genome and their environment, including physical activity level, diet, and other stressors. Therefore, proteome studies are well placed to provide the next generation of clinical biomarkers.

While the potential fruits of proteomics are great, so are the challenges. Not only is the proteome more expansive than any other biological level, but also proteins exhibit a broad range of different physicochemical properties, which affect their solubility and make it difficult to extract all proteins using a single technique. Moreover, the dynamic range of protein abundance spans at least 7 orders of magnitude [2], and the proteins cannot be amplified and the entire proteome is not known. In particular, proteoforms cannot be predicted and must be identified and characterised empirically, and cataloguing of proteins using mining techniques (e.g., [3, 4]) is an important aspect of cardiac proteomics work. Unlike nucleic acids, interaction between proteins is not based on well-understood complimentary binding so it is difficult to predict protein-protein interactions, binding partners, substrates, and so forth. To address these and other challenges, different proteomic techniques have been developed for descriptive and comparative studies. Nonetheless, proteomic studies share common building blocks including proteome separation, mass spectrometry analysis, and protein identification (Figure 1).

Conceptually, proteomic investigations are described as either “bottom-up” or “top-down.” Top-down studies such as those of S. Yavuz et al. and D. Zheng et al. typically employ 2-dimensional gel electrophoresis (2DGE) to spatially separate proteins according to their isoelectric point (p*I*; first dimension, isoelectric focusing) and relative molecular mass (*M*_*r*_; second dimension, denaturing gel electrophoresis). Image analysis is used to discover differences in spot volumes between case and control samples. Then, gel spots are excised and the proteins digested with trypsin and analysed by mass spectrometry to identify the proteins of interest. Hence, the workflow begins at the protein level and works down to the peptide level, that is, top-down. This sequence also illustrates the open/discovery philosophy of proteomics, wherein differences between samples are detected before the protein is identified, which is diametrically opposite to hypothesis-led reductionist designs where the target of interest must be defined* a priori*.

S. Yavuz et al. report 2DGE analysis of pericardial fluid and highlight omentin-1 as a candidate biomarker that may assist in the classification of pericarditis. Currently, more than one-quarter of pericarditis cases are designated as idiopathic based on lack of classification by current biochemical techniques. Mining of pericardial fluid proteome has been described [5] but few other studies report comparative analysis of this important body fluid. D. Zheng et al. also address the need for biomarkers directly in human clinical samples using a top-down approach. Rheumatic heart disease, which is a serious problem in developing countries, is difficult to diagnose because patients often do not present typical cardiovascular risk factors. D. Zheng et al. used 2DGE to show that cardiac samples of rheumatic heart disease have a drastically greater abundance of heat shock protein 60 compared to matched samples from patients with mitral valve prolapse. What is evident from each of these studies is that the novel biomarkers fit well with our current mechanistic understanding of these diseases.

While 2DGE offers robust comparative analysis of protein species, standard gel systems have limited ability to resolve proteins larger than ~150 kDa or proteins at the extremes of the p*I* scale (i.e., <pH 4 or >pH 9). Bottom-up workflows overcome these issues by digesting the sample into peptides, which have fewer solubility issues. Typically, peptides are resolved using reverse phase liquid chromatography (RPLC) and tandem mass spectrometry, which records peptide ion masses and their fragment-ion spectra. Using this approach, differences between case and control are determined based on mass spectrometry data of either the relative intensity (ion abundance) or number (spectral counting) of the peptide ions. Alternatively, samples can be labelled using differential mass-tags, which enables groups of samples to be combined and analysed equivalently. The major challenge in this workflow lies in the data analysis. In particular, peptides must be unambiguously linked to their parent protein and, ideally, isoform-specific peptides should be used for quantitation; otherwise, differences must be described for the group of proteins to which those peptides could belong.

S. Abdul-Ghani et al. report cardiac phosphoproteome responses to remote ischaemic preconditioning (RIPC) in mice using a peptide labeling bottom-up technique. The protective effects of RIPC have been convincingly demonstrated but the underlying mechanisms have yet to be identified (reviewed in [6]). Phosphopeptide analysis is particularly challenging because the number of phosphorylated copies of a protein is usually a relatively small proportion of the total. Therefore, selective enrichment strategies such as TiO_2_ affinity are used. This strategy enabled S. Abdul-Ghani et al. to discover a novel site-specific phosphorylation of the Z-disc protein, myozenin-2, which may have a signalling role in RIPC cardioprotection. L. E. de Castro Brás et al. also report the use of a bottom-up technique and investigated age-associated differences in the extracellular matrix composition of mice lacking the SPARC gene. SPARC (secreted protein acidic and rich in cysteine) is a matricellular protein involved in the assembly of collagen fibrils. Loss or gain of the function of SPARC impacts cardiac fibrosis, and L. E. de Castro Brás et al. used orthogonal separation processes (SDS-PAGE and RPLC) and spectral counting to reveal that SPARC may contribute to age-associated cardiac stiffening.

In addition to the aforementioned original research articles, this special issue includes comprehensive reviews of hypertension- and exercise-related cardiac adaptations, presented by B. A. Petriz and O. L. Franco, and dystrophinopathy-associated cardiomyopathy, presented by A. Holland and K. Ohlendieck. These reviews bracket the continuum spanning from monogenic disease to complex polygenic traits and highlight how proteomic strategies are being used to advance these fields. We hope this special issue provides readers with new insight to cardiac research and the role that proteomics can play in generating novel information and candidate biomarkers.

*Jatin G. Burniston* *Anthony O. Gramolini* *R. John Solaro*

## Figures and Tables

**Figure 1:**
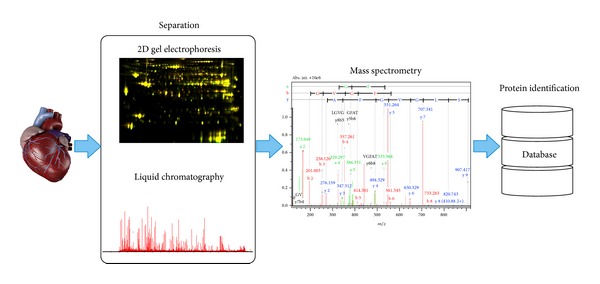
Proteomic workflow. Proteomic experiments share a common workflow encompassing separation, mass analysis, and protein identification. The top-down approach uses image analysis of proteins separated by 2-dimensional gel electrophoresis to find differences between case and control samples. Protein spots of interest are then identified by comparing their peptide mass spectrometry data to protein databases. Conversely, bottom-up workflows first digest proteins extracted from cardiac samples into peptides. The peptides are separated by reverse-phase liquid chromatography, and mass spectrometry is used to identify and quantify the proteins. Common to each of these strategies is the philosophy that differential analysis is performed prior to protein identification. This purposeful “open” approach enables proteomic work to discover information that might not otherwise have been hypothesized.
